# In Situ Incorporation of Diamino Silane Group into Waterborne Polyurethane for Enhancing Surface Hydrophobicity of Coating

**DOI:** 10.3390/molecules24091667

**Published:** 2019-04-28

**Authors:** Jinghui Lyu, Kaiyue Xu, Ning Zhang, Chunshan Lu, Qunfeng Zhang, Lu Yu, Feng Feng, Xiaonian Li

**Affiliations:** State Key Laboratory Breeding Base of Green Chemistry Synthesis Technology, College of Chemical Engineering, Zhejiang University of Technology, Hangzhou 310032, China; lyujh@zjut.edu.cn (J.L.); 13386529829@163.com (K.X.); 19857106774@163.com (N.Z.); Zhangqf@zjut.edu.cn (Q.Z.); yuluuni2014@gmail.com (L.Y.); ffeng@zjut.edu.cn (F.F.); xnli@zjut.edu.cn (X.L.)

**Keywords:** waterborne polyurethane, cross-linked siloxane, APTS

## Abstract

A series of waterborne polyurethanes (WPU) with crosslinked siloxane were obtained through introducing 3-(2-aminoethylamino)propyldimethoxymethylsilane (APTS) into WPU by in situ polymerization. The properties of WPU modified by APTS were studied through a variety of experimental methods. The water contact angle of the WPU coating surface increased from 64° to 86°, and the water resistance reduced to 3.90% when 3 wt% APTS was added, which improved the coating surface hydrophobicity. Firstly, Fourier transform infrared (FT-IR) and ^1^H-NMR spectra demonstrated the successful incorporation of APTS to polyurethanes and completed the hydrolytic condensation reaction-generated Si–O–Si crosslinking structure. Furthermore, the surface energy of the membrane was reduced when the crosslinking structure migrated and enriched on the surface of film. Besides, the crosslinking structure was abundant, and the distribution of siloxane in WPU was more uniform.

## 1. Introduction

An eco-friendly material, waterborne polyurethane (WPU), has now been gradually replacing the traditional solvent polyurethane due to its use of safe, non-toxic, non-combustible water as dispersant [1,2]. Refitting from the inherent properties of polyurethane materials, WPU can be flexibly designed to meet the requirements of different industries by changing its molecular weight, chemical composition, and molar ratio of soft and hard segments. As one of the most studied materials, WPU has been widely utilized in various manufacturing industries to produce coatings [3,4], foams [5,6,7], adhesives [8,9,10], and elastomers [11], etc. However, most of the WPU materials are one-component linear thermoplastic polymers with hydrophilic ionic groups in the backbone of WPU. Furthermore, the crosslinking density of WPU has to be kept low in order to obtain good dispersion stability in the water phase, which greatly lowered the adhesive properties, water resistance, and thermal stability of WPU, causing it to be less competitive in the practical application in contrast to the conventional organic solvent-based polyurethane [12]. 

Siloxane is an important additive to modify WPU, since its inorganic Si–O bond in silane results in low surface energy, good thermal stability, and excellent flexibility, which made it the perfect candidate for WPU modification [13,14]. Moreover, the alkoxy groups in silane at the end of the molecular chains can be hydrolyzed to form silanol groups, which enables condensation reactions to occur between these silanol groups to form Si–O–Si crosslinks [15,16]. In addition, during WPU curing, non-polar silicon chains tend to migrate and accumulate on the surface of WPU emulsions [17], resulting in the low surface tension of WPU film. Furthermore, siloxane-modified WPU has better heat resistance and low temperature elasticity than pure WPU [18,19]. Silane has already been successfully incorporated into polyurethanes by several independent research groups via amino groups [20] and hydroxyl groups [21,22].

In most methods reported for improving the properties of WPU siloxane when introduced at its two chain ends, the entrained Si–O–R provides the modified polyurethane with a potential of being crosslinked, which may improve its water resistance, surface hydrophilicity, and mechanical properties [23]. However, siloxane can be only located at the two ends of WPU, which would not only limit the content of siloxane, but also make the number of crosslinks small. The properties of the polyurethane may be improved, but only to a limited extent. 

In order to increase the content and distribution of siloxane on WPU, we synthesized a series of WPU modified by chain extender 3-(2-aminoethylamino)propyldimethoxymethylsilane (APTS), which contain two amines, two methoxysilanes, and one methyl group. Siloxane is introduced into the WPU side chain by connecting the molecules of the two amino groups with the PU prepolymer, making it evenly distributed on the PU side chain, not just only at both ends of the PU chain. Even a small amount of APTS would increase the content and distribution of siloxane on the WPU.

## 2. Materials and Methods

### 2.1. Materials

Isophorone diisocyanate (IPDI), dimethylacetamide (DMAc), triethylamine (TEA), acetone, 3-(2-aminoethylamino)propyldimethoxymethylsilane (APTS), 2,2-dimethylolpropionic acid (DMPA), and dibutyltin dilaurate (DBTDL) were all purchased from Aladdin (Shanghai, China). Polycarbonate diols (PCDL, Mn = 2000) was purchased from Jining huakai resin (Jining, China). PCDL and DMPA were dried at 120 °C under vacuum for 2 h to remove any residual water before use. Other reagents were used directly without purification. Deionized water was prepared in the laboratory, and was used as the dispersing phase.

### 2.2. Experiments

#### 2.2.1. Synthesis of APTS-Modified Waterborne Polyurethane Emulsion

**Figure 1:**
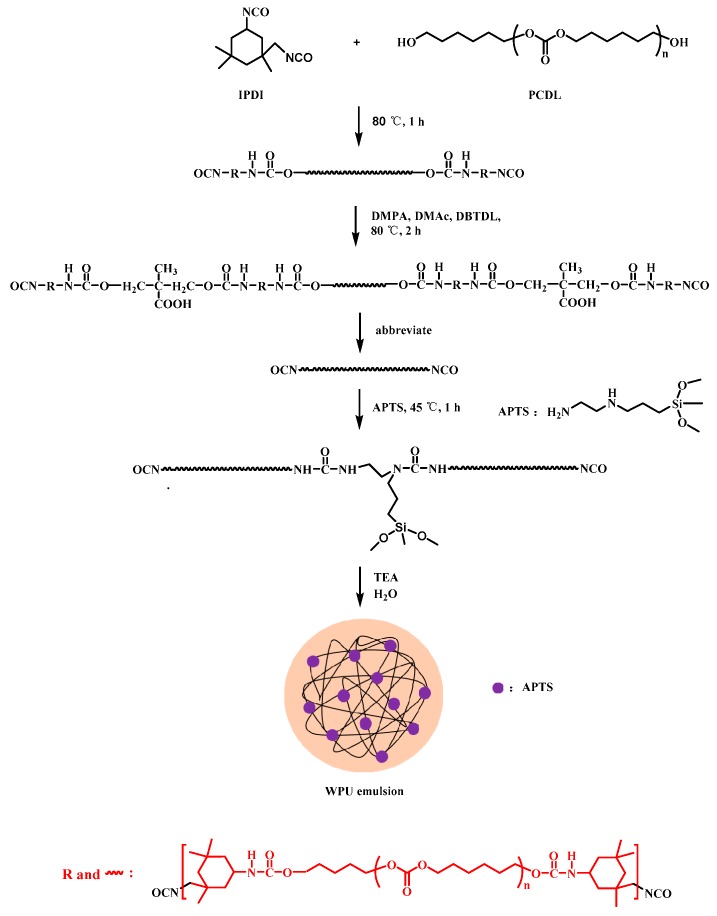
The synthesis process of waterborne polyurethanes (WPU) modified with APTS.

A 500-mL four-neck glass reactor with a mechanical stirrer, thermometer, condenser tube, nitrogen inlet, and outlet was used. IPDI and PCDL of certain amounts were added into the reactor, and the reaction was conducted in a water bath maintained at 80 °C for 1.5 h. Then, DMPA, DMAc, and DBTDL were added. After another 2 h of reaction at 80 °C, the –NCO terminated prepolymer was obtained. The prepolymer was cooled to 45 °C, and APTS was added to react with it for chain extension. After reacting for 1 h, TEA was added to neutralize the reaction mixture. Acetone was added during the reaction to reduce the viscosity. After 30 min, the neutralized reaction mixture was dispersed in distilled water under vigorous stirring for 1 h. The resulting product was APTS-modified WPU emulsion with a solid content of about 37%. Acetone was removed from the above dispersion under reduced pressure at 30 °C. The preparation process for WPU is illustrated in Figure 1, and the compositions of all the samples are given in Table 1.

#### 2.2.2. Preparation of WPU Films

All the emulsions were smeared evenly on a polytetrafluoroethylene mold at room temperature for 3–4 days for moisture-induced curing. Then, the films were dried in a drying oven at 80 °C until two consecutive weighing errors were less than 0.01.

### 2.3. Characterization

Fourier transform infrared (FT-IR) spectroscopy analysis: The FT-IR spectra of WPU was finished by a Nicolet 6700 Fourier transform infrared spectrometer (Thermo, Waltham, MA, USA) ranging from 4000 cm^−1^ to 600 cm^−1^.

The ^1^H-NMR (500 MHz) spectra: The ^1^H-NMR (500 MHz) spectra of WPU were recorded in deuterated dimethyl sulfoxide (DMSO) solution using a Bruker AVANCE 500 MHz fully digitized Fourier superconducting NMR spectrometer (Bruker, Bern, Switzerland).

The molecular weight analysis: The average molecular weights of the WPU chain were measured using Malvern Viscotek DPC-270 (Malvern, London, UK). These experiments were performed at 35 °C in chromatography grade chloroform using PLgel5 Mixed-C columns at a flow rate of 1 mL/min. Conventional calibrations were performed using polystyrene standards.

The average particle size analysis: The average particle size of WPU emulsion was measured with a Malvern Nano-ZS laser particle sizer (Malvern, London, UK).

Differential Scanning Calorimetry analysis: The thermal property of WPU was measured using a TA Instruments Q-20 (TA Instruments, New Castle, DE, USA) differential scanning calorimeter analyzer. First, 3~8 mg WPU films were films hermetically sealed in an aluminum pan were heated up to 80 °C with a heating rate of 20 °C/min and kept for 3 min to keep a consistent thermal history for the melting process. Then, the samples were cooled to −80 °C at a cool rate of 20 °C/min. The non-isothermal measurement was scanned from −80 to 80 °C with a heating rate of 20 °C/min.

Scanning electron microscopy analysis: The surface morphology of the WPU samples was analyzed using scanning electron microscopy HitachiS-4700 (JEOL, Tokyo, Japan) at an accelerating voltage of 25 kV. Samples were adhered to aluminum sample holders and sputter coated with Au layers.

Atomic force microscopy analysis: Atomic force microscopy (AFM) measurements were performed on the instrument CSPM-2003 (Original nanometer instruments, Beijing, China) with a 5 µm × 5 mm scan area, and images were acquired under ambient conditions in tapping mode using a nanoprobe cantilever.

Thermogravimetric analysis: TA-Q5000IR (TA Instruments, New Castle, DE, USA) was used to monitor the thermal decomposition process of the WPU films. The temperature range was 100 to 700 °C in nitrogen atmosphere, and the heating rate was 10 °C/min.

Shore A Hardness Measurements: The Shore A hardness of WPU films were measured by LX-A Shore Rubber Hardness (Huayin Instrument, Zhengzhou, China), and the test method was GB 2411-80. Each hardness measurement was repeated five times.

Mechanical testing: At room temperature, the tensile properties of the WPU films was tested by using the AI-7000SGD Taiwan high iron tensile testing machine (High iron equipment testing company, Hangzhou, China) at a speed of 50 mm/min. The test was performed on five samples of each film to take an average for the report.

Static contact angle measurements: The static contact angles of deionized water on the surface of WPU films were measured by a sessile drop method with a Dataphysics OCA30 contact angle goniometer (Dataphysics, Berlin, Germany) at 25 °C. The results displayed were the average values of five specimens.

Water absorption measurements: The film was cut into small pieces with a size of 25 cm × 25 cm at room temperature; the initial weight W_1_ was taken before the small piece of sample was immersed in deionized water for 72 h. Then, when the sample was taken out of the deionized water, it was quickly dried to remove the surface moisture and weighed to obtain the W_2_. The water absorption of membrane was calculated using the following equation:

(1) $$\mathrm{water}\;\mathrm{absorption}\left(\%\right)=\frac{W_{1}-W_{2}}{W_{1}}\times 100\%$$

## 3. Results

### 3.1. Characterization of WPU

**Figure 2:**
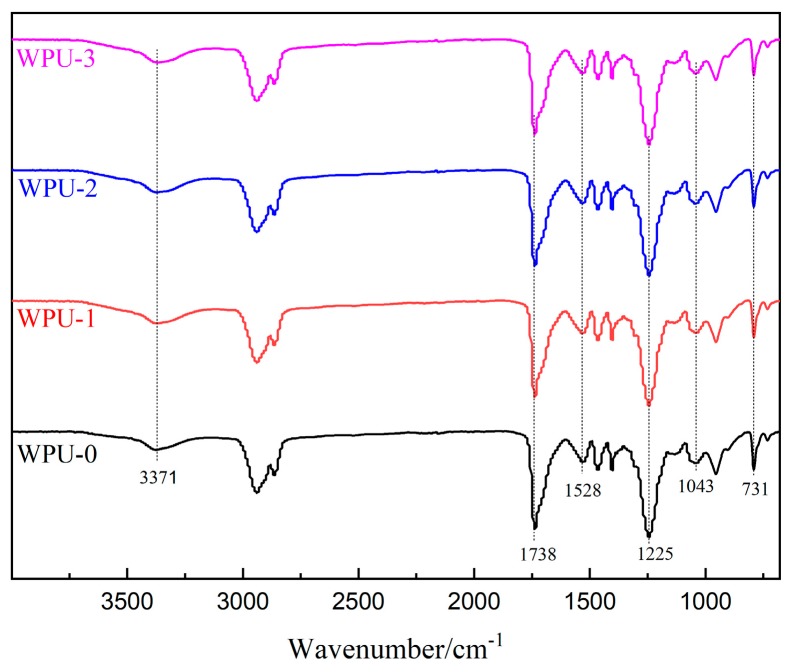
Infrared spectra of different WPU.

As shown in Figure 2, there is no obvious difference between the FT-IR spectra of different WPU films. The peaks at 3371 cm^−1^ and 1737 cm^−1^ corresponding to the stretching vibration of the N–H bond and C=O bond, and the peak at 1528 cm^−1^ originated from the bending vibration of N–H bond, implied the formation of urethane groups. The absorption bands at 2862–2939 cm^−1^ are ascribed to the stretching vibration movement of the C–H bond from the CH_2_ and CH_3_ groups. The absorption band at 1225 cm^−1^ is ascribed to the C=O of the polycarbonate glycol. For WPU-1, WPU-2, and WPU-3, the absorption peaks for the stretching vibration of Si–O–Si and C–O–C were at 1043 cm^−1^ and 731 cm^−1^ [24]. 

**Figure 3:**
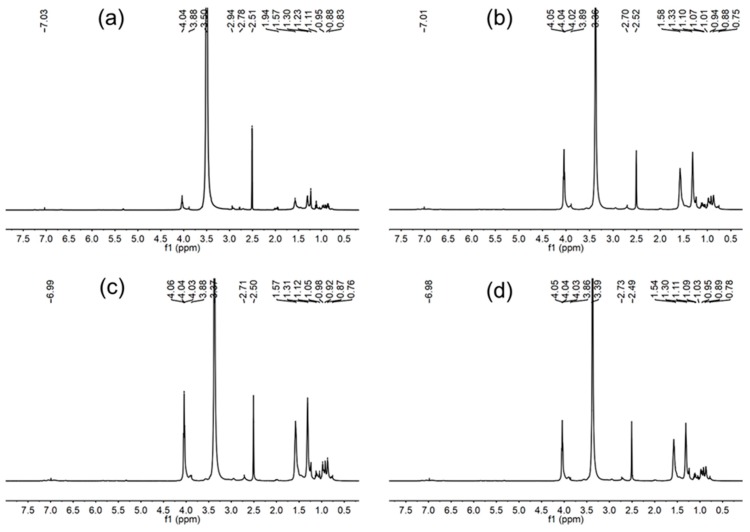
^1^H-NMR spectra of different WPU: (**a**) WPU-0; (**b**) WPU-1; (**c**) WPU-2; and (**d**) WPU-3.

^1^H-NMR spectrum of WPU-0 (Figure 3a) illustrated the presence of the N–H proton from the urethane groups (6.98~7.03 ppm). The observed peaks at 3.88 to 4.04 ppm and 0.83 to 0.95 ppm were assigned to the protons of the urethane –CH_2_– group from DMPA and methyl protons from IPDI, respectively. The methylene protons of IPDI and methyl protons of DMPA were observed at 0.95~1.11 ppm. The singlet at 3.50 ppm was assigned to the –CH_2_– group from PCDL. 

Figure 3b–d is the ^1^H-NMR spectrum of polyurethane with different content of APTS; the CH_2_ protons attached to the NH_2_ group of APTS and silicon atom are observed around at the 2.71 ppm and 0.76 ppm peaks, respectively. The signals of the Si–OH (at δ = 5.0) and methyl protons of Si–O–CH_3_ (at δ = 3.50) were absent, which indicated that hydrolysis and condensation reaction of Si–O–CH_3_ were completed. From the FT-IR and ^1^H-NMR results, we concluded that the polyurethane had incorporated with APTS successfully.

### 3.2. Molecular Weight of WPU Emulsion

Table 2 shows that the addition of increasing amounts of APTS (from 0 wt% for WPU-0 to 3 wt% for WPU-3) causes an increase in the molecular weight of WPU accordingly (from 2.59 × 10^4^ g/mol for WPU-0 to 5.13 × 10^4^ g/mol for WPU-3). We might infer that the chain length is controlled with the addition content of APTS. Since the role of APTS in the reaction is that of a chain extender [25], the result in Table 2 thus demonstrated the successful attachment of APTS on the WPU system, which resulted in the rise of the molecular weight of WPU [26]. 

### 3.3. Particle Size of WPU Emulsion

**Figure 4:**
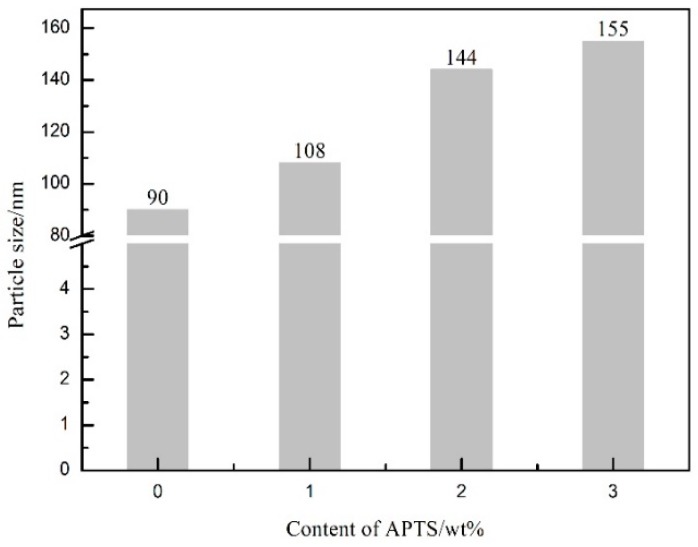
The average particle size of the different WPU emulsions.

Figure 4 shows the average particle size of WPU emulsions modified by different amounts of APTS. As can be seen from the Figure 4, the average particle size of WPU was highly dependent on the APTS content. As the APTS content increased from 0 to 3.0 wt%, the average diameter of WPU emulsions gradually rose from 90 to 155 nm. During the investigation, other factors proved to have an effect on the particle size, such as the mole ratio of NCO/OH. The hydrophilic group, neutralization, and emulsification conditions [27] were all excluded to make sure that the difference detected originated solely from the effect of the chain extender. In the synthesis process, the IPDI, PCDL, and DMPA were the same in each experiment, and the content of NCO/OH and the hydrophilic groups were guaranteed to be the same as well. The only variable in the experiment was the content of APTS. APTS is a chain extender; it can increase the molecular weight of PU polymers when it is attached to them [28]. In addition, an increase in APTS content will insert more hydrophobic structure into the polymer chains [29], and the formation of Si–O–Si linkages increases the branched structure [30]. Therefore, the average particle size of WPU emulsions gradually increases with elevated APTS content.

### 3.4. Thermal Properties of the WPU Films

**Figure 5:**
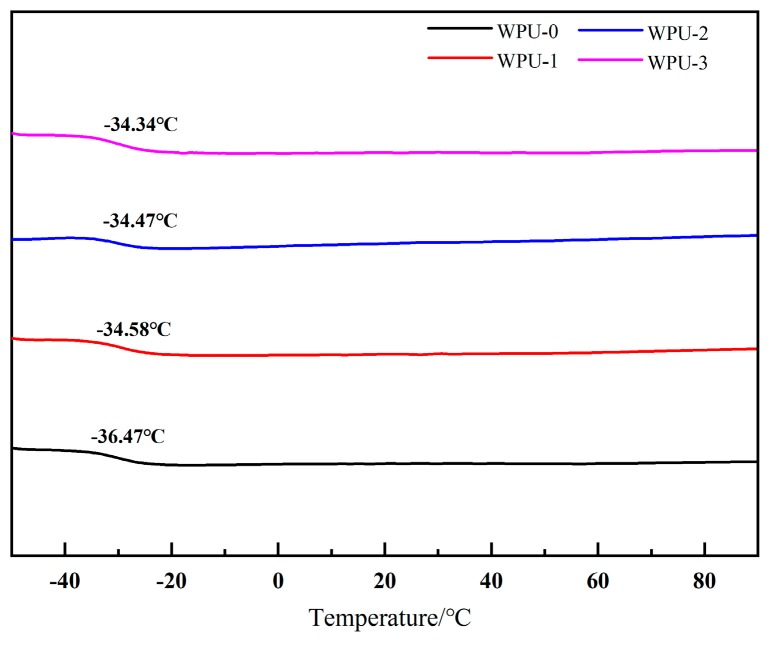
Differential scanning calorimetry (DSC) thermograms of WPU dispersions with different APTS contents.

The glass transition temperatures of all the samples were measured by differential scanning calorimetry (DSC). As the amount of APTS added increased from 0 to 3wt%, the glass transition temperature of the soft segment increased from −36.47 to −34.34 °C (Figure 5), which was a result of the increased crosslinking degree [31].

**Figure 6:**
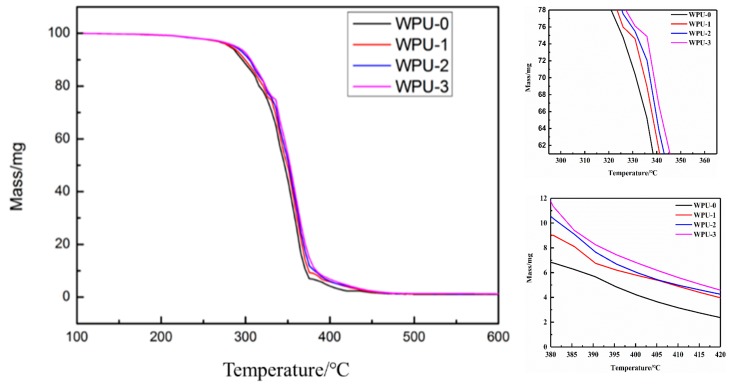
The TG curve of different WPU films.

Figure 6 shows the TG (relationship between mass change and temperature) curve of different WPU films; Table 3 shows the thermal decomposition temperatures of the first (T_max1_) and second (T_max2_) stages of different WPU films. The thermal decomposition process of the polyurethane film is divided into two stages, which are the thermal decomposition of the hard (carbamate group and the urea bond) and soft (polyester) segments, namely [32]. At low temperature (around 250 °C), the mass loss was mainly due to the volatilization of organic solvents and water remaining in the polyurethane film [33]. Carbamate decomposition occurred in the range of 250 to 360 °C, which was indicated by the rapid mass loss due to the formation of isocyanates, alcohols, and amines [34]. While at higher temperature (above 390 °C), the fracture of the polyester and the crosslinked structure were the major causes of mass loss.

When the APTS content was increased from 0 to 3 wt%, the first degradation temperature was increased from 324 to 335 °C, and the second degradation temperature was increased from 395 to 415 °C. This enhancement in the thermal stability of the WPU film caused by upraised APTS content was rationalized to the higher bond energy of Si–O (460 kJ/mol) than C–C (326 kJ/mol), and the formation of the Si–O–Si crosslinked network, which increased the interaction between the molecular chains and prevented the evaporation of the segment [35].

### 3.5. Mechanical Properties of the WPU Films

**Figure 7:**
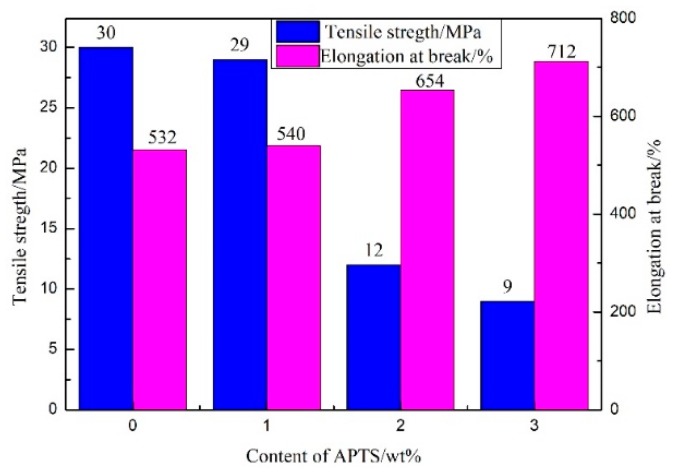
Effect of APTS on the mechanical properties of WPU films.

As shown in Figure 7, the tensile strength of the WPU films decreased, and the elongation at the break increased with the increase in APTS content. The PCDL contained a large amount of carbonate bonds and ether bonds that could easily form hydrogen bonds with the hard segment of WPU films (carbamate groups). However, for the APTS-modified PCDL type of WPU, the formation of intermolecular hydrogen bonds was inhibited by the sterical hindrance originated from the large, low-polarity APTS segments attached on the side chain [36]. The tensile strength of PCDL depends mainly on the intramolecular and intermolecular hydrogen bonding [37]. Thus, it causes a negative correlation between the amount of APTS and the tensile strength of the WPU film.

WPU is a block copolymer that is composed of alternated hard segments and soft segments in its molecular chains [38]. As mentioned before, the Si–O–Si crosslinked network structure formed by the addition of APTS belongs to the soft segment [39,40]. Therefore, when the APTS content was increased, the percentage composition of the crosslinking structure and thus the soft segments in the WPU films will be increased, and hence ‘soften’ the WPU films [41]. Table 2 shows that when the APTS content increased from 0 to 3 wt%, the shore hardness decreased from 1.60 to 1.38 N, indicating that the hardness of the waterborne polyurethane film decreased and became softer. Therefore, when the APTS content increased from 0 to 3 wt%, the breaking elongation increased from 532% to 712%. The tensile strength and elongation at break of the WPU film modified by Zhao et al. with bis-amino-containing siloxane were 6.26 MPa [17]. In our study, the tensile strength and elongation at break were 9 MPa; the mechanical properties are significantly higher than those of Zhao et al. (Table S3). 

### 3.6. Surface Property and Water Absorption of WPU Films

**Figure 8:**
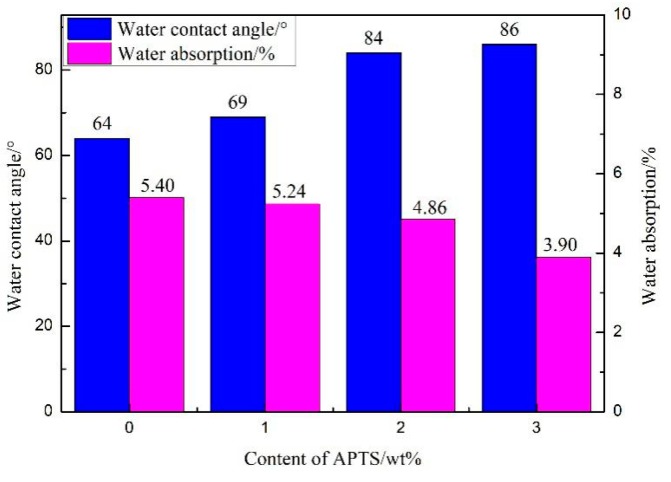
Water contact angles and water absorption of different WPU films.

The hydrophobicity of the different content of APTS-modified WPU films was investigated by measuring their static contact angles (Figure 8). It was found that with the increase of APTS content from 0 to 3 wt%, the contact angle of the water on the WPU film increased from 64° to 86°. This is primarily caused by the Si–O–Si network structure [42,43]. During the film formation and crosslinking process, the presence of siloxane with low polarity can provide a thermodynamic driving force for its surface migration, decrease the surface energy, and make the surface hydrophobic [44], which leads to the observation shown in Figure 8. In the studies by Zhao et al. [17] and Lei et al. [29], the maximum water contact angle reached respectively 79.25° and 77.6°. In our study, the amount of APTS added was small, and the hydrophobicity of the modified WPU film was relatively good (Tables S2 and S3).

The addition of APTS into the PU systems can improve the water resistance of WPU films, since WPU films with higher APTS content have lower water absorption capacity (Figure 8). The surface properties and water absorption capacities of the WPU films examined here in this study were significantly higher than those reported by Lei et al. [29]. Apart from the presence of hydrophobic Si–O–Si groups in the crosslinked structure described before, the tendency of highly crosslinked Si–O–Si structures to migrate toward the membrane surface and thus reduce water infiltration was also responsible for the decrease in the water absorption capacity of the films with high APTS content [45]. 

## 4. Conclusions

In this work, siloxane-modified WPU emulsion was synthesized by introducing APTS into WPU by in situ polymerization. FT-IR and ^1^H-NMR spectra indicated that APTS has been introduced into polyurethane successfully, and hydrolyzed condensation reaction was completed. The Si–O–Si crosslinking structure was strengthened, and the crosslinking density of WPU increased with a small amount of APTS introduced, which greatly improved the properties of WPU, especially its hydrophobic property and water resistance. As the APTS content increased from 0 to 3 wt%, the average particle size of WPU emulsion increased from 90 to 155 nm, the water contact angle increased from 64° to 86°, and the water absorption reduced from 5.40% to 3.90%. The TG and tensile experiment indicated that the Si–O–Si crosslinking structure can improve the thermodynamic properties and increase the flexibility of the film. Hence, the WPU coating with crosslinked siloxane by in situ polymerization would have a good application prospect in waterproof coating.

## Figures and Tables

**Table 1:** Molar ratio of raw materials for the synthesis of WPU. IPDI: isophorone diisocyanate, PCDL: polycarbonate diols, DMPA: 2,2-dimethylolpropionic acid, TEA: triethylamine (TEA), APTS: 3-(2-aminoethylamino)propyldimethoxymethylsilane.

	IPDI (mol)	PCDL (mol)	DMPA (mol)	TEA (mol)	APTS (mol)	APTS (wt%)
WPU-0	0.0605	0.0235	0.0265	0.0265	0	0
WPU-1	0.0605	0.0235	0.0265	0.0265	0.0005	1
WPU-2	0.0605	0.0235	0.0265	0.0265	0.001	2
WPU-3	0.0605	0.0235	0.0265	0.0265	0.015	3

**Table 2:** Molecular weight of WPU samples prepared using different amount of APTS.

Sample	WPU-0	WPU-1	WPU-2	WPU-3
Mn (g/mol)	2.59 × 10^4^	4.32 × 10^4^	4.68 × 10^4^	5.13 × 10^4^

**Table 3:** Properties of WPU emulsions and films.

		WPU-0	WPU-1	WPU-2	WPU-3
TG (℃)	T_max1_	324	330	331	335
	T_max2_	395	405	410	415
Shore Hardness (N)		1.60	1.56	1.45	1.38

## Supplementary Materials

The following are available online, Figure S1: SEM micrographs of the surfaces of WPU films (scale bar = 5 µm), Figure S2: 3D AFM height images of WPU films modified with APTS, Table S1: Surface roughness of WPU films modified with APTS, Table S2: Comparison of the water contact angle, Table S3: Comparison of the tensile strength.

## References

[1] Feng J.Y., Wang X.C., Guo P.Y., Wang Y.J., Luo X.M. (2018). Mechanical Properties and Wear Resistance of Sulfonated Graphene/Waterborne Polyurethane Composites Prepared by In Situ Method. Polymers.

[2] Kang S.Y., Ji Z.X., Tseng L.F., Turner S.A., Villanueva D.A., Johnson R., Albano A., Langer R. (2018). Design and Synthesis of Waterborne Polyurethanes. Adv. Mater..

[3] Yin X., Lin X.Y., Luo Y.J. (2017). Synthesis and Characterization of Multifunctional Two-Component Waterborne Polyurethane Coatings: Fluorescence, Thermostability and Flame Retardancy. Polymers.

[4] Wang F.F., Feng L.J., Li G.Z. (2018). Properties of Waterborne Polyurethane Conductive Coating with Low MWCNTs Content by Electrostatic Spraying. Polymers.

[5] Chen H.B., Shen P., Chen M.J., Zhao H.B., Schiraldi D.A. (2016). Highly Efficient Flame Retardant Polyurethane Foam with Alginate/Clay Aerogel Coating. Appl. Mater. Inter..

[6] Fang Z., Yang Z., Ji D., Zhu N., Li X., Wan L., Zhang K., Guo K. (2016). Novel Synthesis of a Soy-based Polyol for a Polyurethane Rigid Foam. RSC Adv..

[7] Hassan A., Rashid Y., Mourad A.H.I., Ismail N., Laghari M.S. (2019). Thermal and Structural Characterization of Geopolymer-Coated Polyurethane Foam—Phase Change Material Capsules/Geopolymer Concrete Composites. Materials.

[8] Cakić S.M., Ristić I.S., M.-Cincović M., Stojiljković D.T., B.-Simendić J. (2016). Preparation and Characterization of Waterborne Polyurethane/silica Hybrid Dispersions from Castor oil Polyols Obtained by Glycolysis Poly(ethylene terephthalate) Waste. Int. J. Adhes. Adhes..

[9] Che J.Y., Cheon J.M., Chun J.H., Park C.C., Lee Y.H., Kim H.D. (2017). Preparation and Properties of Emulsifier-/Solvent-free Slightly Crosslinked Waterborne Polyurethane-acrylic Hybrid Emulsions for Footwear Adhesives (III)–effect of Trimethylol Propane (TMP)/Ethylene Diamine (EDA) Content. J. Adhes. Sci. Technol..

[10] Rahman M., Zahir M., Kim H. (2016). Synthesis and Properties of Waterborne Polyurethane (WBPU)/Modified Lignin Amine (MLA) Adhesive: A Promising Adhesive Material. Polymers.

[11] Behera P.K., Usha K.M., Guchhait P.K., Jehnichen D., Das A., Voit B., Singha N.K. (2016). A Novel Ionomeric Polyurethane Elastomer Based on Ionic Liquid as Crosslinker. RSC Adv..

[12] Lei L., Zhong L., Lin X.Q., Li Y.Y., Xia Z. (2014). Synthesis and Characterization of Waterborne Polyurethane Dispersions with Different Chain Extenders for Potential Application in Waterborne Ink. Chem. Eng. J..

[13] Zheng G.K., Lu M., Rui X.P., Shao B. (2018). Surface and Bulk Properties of Waterborne Polyurethane Modified with Fluorinated Siloxane. J. Appl. Polym. Sci..

[14] Han Y., Hu J., Xin Z. (2018). In-Situ Incorporation of Alkyl-Grafted Silica into Waterborne Polyurethane with High Solid Content for Enhanced Physical Properties of Coatings. Polymers.

[15] Sardon H., Irusta L., Fernández-Berridi M.J., Lansalot M., Bourgeat-Lami E. (2010). Synthesis of Room Temperature Self-curable Waterborne Hybrid Polyurethanes Functionalized with (3-aminopropyl)triethoxysilane (APTES). Polymer.

[16] Zhai L.L., Liu R.W., Peng F., Zhang Y.H., Zhong K., Yuan J.X., Lan Y.J. (2012). Synthesis and Characterization of Nanosilica/Waterborne Polyurethane End-capped by Alkoxysilane via a Sol-gel Process. J. Appl. Polym. Sci..

[17] Zhao H., Huang D., Hao T.H., Hu G.H., Ye G.B., Jiang T., Zhang Q.C. (2017). Synthesis and investigation of well-defined silane terminated and segmented waterborne hybrid polyurethanes. New J. Chem..

[18] Subramani S., Lee J.M., Cheong I.W., Kim J.H. (2005). Synthesis and Characterization of Water-borne Crosslinked Silylated Polyurethane Dispersions. J. Appl. Polym. Sci..

[19] Fei G.Q., Shen Y.D., Wang H.H., Shen Y. (2006). Effects of Polydimethylsiloxane Concentration on Properties of Polyurethane/Polydimethylsiloxane Hybrid Dispersions. J. Appl. Polym. Sci..

[20] Wu Y., Guo P., Zhao Y., Liu X.J., Du Z.L. (2019). Hydrophobic, Transparent Waterborne Polyurethane-polydimethylsiloxane Composites Prepared From Aqueous Sol-gel Process and Applied in Corrosion Protection. Prog. Org. Coat..

[21] Wang G., Ma G.Z., Hou C.Y., Guan T.T., Ling L.X., Wang B.J. (2014). Preparation and Properties of Waterborne Polyurethane/Nanosilica Composites: A Diol as Extender with Triethoxysilane Group. J. Appl. Polym. Sci..

[22] Shaik A., Narayan R., Raju K.V.S.N. (2014). Synthesis and Properties of Siloxane-crosslinked Polyurethane-urea/Silica Hybrid Films from Castor Oil. J. Coat. Technol. Res..

[23] Li Q., Ye J., Qiu T., Guo L.H., He L., Li X.Y. (2018). Synthesis of Waterborne Polyurethane Containing Alkoxysilane Side Groups: Study on Spacer Linkages. J. Appl. Polym. Sci..

[24] Li X.Y., Hu J., Sun D.X., Zhang Y.H. (2014). Nanosilica Reinforced Waterborne Siloxane-polyurethane Nanocomposites Prepared via “Click” Coupling. J. Coat. Technol. Res..

[25] Xu C.S., OuYang L., Cai Z.S., Ren Y., Lu S.F., Shi W.Z. (2019). Effects of polyaminosiloxane on the structure and properties of modified waterborne polyurethane. J. Appl. Polym. Sci..

[26] Meng X., Shi G.T., Wu C.S., Chen W.J., Xin Z., Shi Y.Q., Sheng Y. (2016). Chain Extension and Oxidation Stabilization of Triphenyl Phosphite (TPP) in PLA. Polym. Degrad. Stab..

[27] Zheng G.K., Lu M., Rui X.P. (2017). The Effect of Polyether Functional Polydimethylsiloxane on Surface and Thermal Properties of Waterborne Polyurethane. Appl. Surf. Sci..

[28] Li Q., Guo L.H., Qiu T., Xiao W.D., Du D.X., Li X.Y. (2016). Synthesis of Waterborne Polyurethane Containing Alkoxysilane Side Groups and the Properties of the Hybrid Coating Films. Appl. Surf. Sci..

[29] Lei L., Zhang Y.H., Ou C.B., Xia Z.B., Zhong L. (2016). Synthesis and Characterization of Waterborne Polyurethanes with Alkoxy Silane Groups in the Side Chains for Potential Application in Waterborne Ink. Prog. Org. Coat..

[30] Yue S.J., Zhang Z.Y., Fan X.J., Liu P., Xiao C.F. (2015). Effect of 3-Aminopropyltriethoxysilane on Solvent Resistance, Thermal Stability, and Mechanical Properties of Two-component Waterborne Polyurethane. Int. J. Polym. Anal. Charact..

[31] Seeni Meera K.M., Murali Sankar R., Jaisankar S.N., Mandal A.B. (2013). Physicochemical Studies on Polyurethane/siloxane Cross-linked Films for Hydrophobic Surfaces by the Sol-gel Process. J. Phys. Chem. B..

[32] Niu Z., Bian F. (2012). Synthesis and Characterization of Multiple Cross-linking UV-curable Waterborne Polyurethane Dispersions. Iran. Polym. J..

[33] Fu H.Q., Wang Y., Li X.Y., Chen W.F. (2016). Synthesis of Vegetable Oil-based Waterborne Polyurethane/Silver-halloysite Antibacterial Nanocomposites. Compos. Sci. Technol..

[34] Petrović Z.S., Yang L., Zlatanić A., Zhang W., Javni I. (2007). Network Structure and Properties of Polyurethanes from Soybean Oil. J. Appl. Polym. Sci..

[35] Cheng Z., Li Q.T., Yan Z., Liao G.F., Zhang B.X., Yu Y.M., Yi C.M., Xu Z.S. (2019). Design and Synthesis of Novel Aminosiloxane Crosslinked Linseed Oil-based Waterborne Polyurethane Composites and its Physicochemical Properties. Prog. Org. Coat..

[36] Li H.L., Liu B.H. (2018). Synthesis and properties of polypropylene carbonate polyol-based waterborne polyurethane modified by KH-560. Nat. Gas Chem. Ind..

[37] Xu C.S., Cai Z.S., Xing J.W., Ren Y., Xu W.Z., Shi W.Z. (2014). Synthesis of Polypropylene Carbonate Polyol-based Waterborne Polyurethane Modified with Polysiloxane and Its Film Properties. Fibers Polym..

[38] Zhou X., Li Y., Fang C.Q., Li S.J., Cheng Y.L., Lei W.Q., Meng X.J. (2015). Recent Advances in Synthesis of Waterborne Polyurethane and Their Application in Water-based Ink: A Review. J. Mater. Sci. Technol..

[39] Zhou H.F., Wang H., Tian X.Y., Zheng K., Cheng Q.T. (2014). Effect of 3-Aminopropyltriethoxysilane on Polycarbonate Based Waterborne Polyurethane Transparent Coatings. Prog. Org. Coat..

[40] Pergal M.V., Džunuzović J.V., Poręba R., Ostojić S., Radulović A., Špírková M. (2013). Microstructure and Properties of Poly(urethane-siloxane)s Based on Hyperbranched Polyester of the Fourth Pseudo Generation. Prog. Org. Coat..

[41] Yu Y.T., Wang J. (2014). Synthesis and Properties of Block and Graft Waterborne Polyurethane Modified with α,ω-*bis*(3-(1-methoxy-2-hydroxypropoxy)propyl)polydimethylsiloxane and α-*N*,*N*-dihydroxyethylaminopropyl-ω-butylpolydimethylsiloxane. Polym. Eng. Sci..

[42] Zhang S.W., Chen Z.D., Guo M., Bai H.Y., Liu X.Y. (2015). Synthesis and Characterization of Waterborne UV-curable Polyurethane Modified with Side-chain Triethoxysilane and Colloidal Silica. Colloids Surf. Physicochem. Eng. Aspects.

[43] Zhang S.W., Guo M., Chen Z.D., Liu Q.H., Liu X.Y. (2014). Grafting Photosensitive Polyurethane onto Colloidal Silica for Use in UV-curing Polyurethane Nanocomposites. Colloids Surf. Physicochem. Eng. Aspects.

[44] Yi T.F., Ma G.Z., Hou C.Y., Li S.S., Zhang R.F., Wu J.B., Hao X.G. (2017). Preparation and Properties of Poly(siloxane-ether-urethane)-acrylic Hybrid Emulsions. J. Appl. Polym. Sci..

[45] Zhao H., Hao T.H., Hu G.H., Shi D., Huang D., Jiang T., Zhang Q.C. (2017). Preparation and Characterization of Polyurethanes with Cross-Linked Siloxane in the Side Chain by Sol-gel Reactions. Materials (Basel).

