# Supplementary material for: In Situ Incorporation of Diamino Silane Group into Waterborne Polyurethane for Enhancing Surface Hydrophobicity of Coating
Source: Molecules. 2019 Apr 28;24(9):1667. doi: 10.3390/molecules24091667 (PMC6539568; doi:10.3390/molecules24091667)
Supplement: Supplementary file 1 [file molecules-24-01667-s001.pdf]

molecules

Supporting Information

1 SEM analysis of the WPU films

Figure S1 shows the surface morphologies of the WPU films characterized using SEM. WPU-0 film displayed rough surfaces. As the APTS content increased, roughness of the film surface decreased. The surfaces of the WPU-3 film were very smooth and were significantly different from films containing lower APTS contents. The microstructures of PU block copolymers can be affected by the chemical composition, block lengths, and thermodynamic miscibility between hard and soft segments. In this work, hard segments were composed of urethane and urea groups and soft segments were composed of polyester carbonyls and Si–O–Si moieties [1, 2]. Moreover, the soft segments were the matrix and the hard ones were dispersed in it. When the concentration of Si element in the film surface was high enough, the domains of hard segments were encapsulated by the soft segments, leading to a smooth surface.

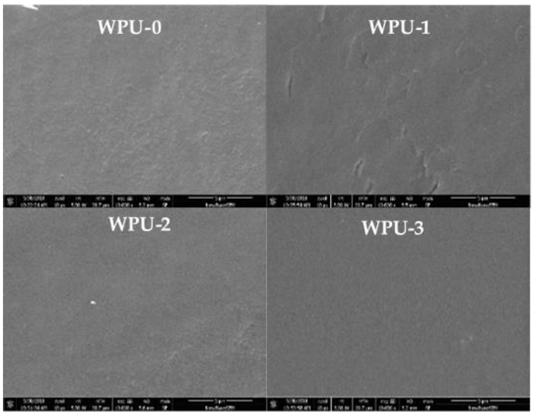

Figure S1. SEM micrographs of the surfaces of WPU films (scale bar = 5 μm)

2 AFM analysis of the WPU films

The surface morphologies of the WPU films were further characterized using AFM. As shown in figure S2, some discontinuous regions with relatively bright and dark domains were observed. The darker part of the "valley" is the soft segment of the PU, which is in continuous phase distribution. And the bright part of the protrusion is the hard section of the PU， which is the dispersed phase [3]. It can be seen from figure S2 that as the APTS content increased, the area of protrusions decreased, indicating that the compatibility of the soft and hard segments is gradually increased, and the phase separation is gradually reduced.

As shown in table S1, the WPU-0 film had a surface irregularity with a root-mean-square surface roughness (Rq) of 15.07 nm, whereas the Rq of WPU-1, WPU-2 and WPU-3 was 9.85 nm, 4.02nm and 1.67 nm respectively, the surface roughness of the film decreased with increasing APTS content, indicating that increasing silane cross-linking imparted smoothness to the film structure.

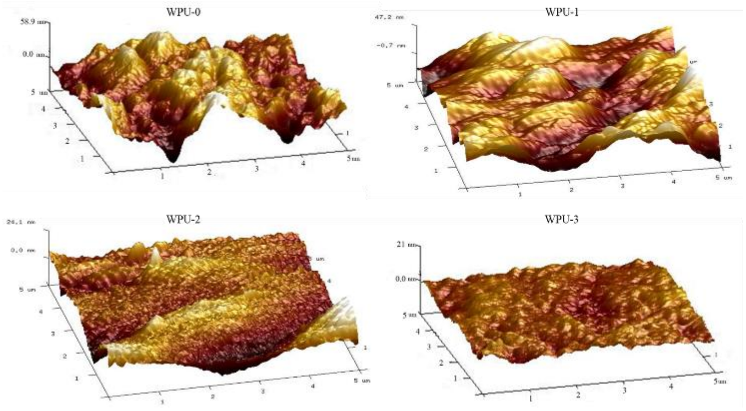

Figure S2. 3D AFM height images of WPU films modified with APTS

Table S1. Surface Roughness of WPU Films Modified with APTS

Sample	Rq (nm)	Ra (nm)	Rmax (nm)
WPU-0	15.07	14.76	40.2
WPU-1	9.85	9.38	24.5
WPU-2	4.02	3.70	16.3
WPU-3	1.67	1.25	13.0

R_q: the root mean square average of height deviations taken from the mean image data plane.

R_a: the arithmetic average of the absolute values of the surface height deviations measured from the mean plane.

R_{max}: the maximum vertical distance between the highest and lowest data points in the image following the planefit.

Table S2. Comparison of the water contact angle

In our study		In the study by Zhao et al [17]		In the study by Lei et al [30]	
reagent content	water contact angle	reagent content	water contact angle	reagent content	water contact angle
0wt%	64 °	0mol%	63.25 °	0wt%	72.5 °
1wt%	69 °	5mol%	72.25 °	1wt%	73.8 °
2wt%	84 °	10mol%	75 °	2wt%	75.8 °
3wt%	86 °	15mol%	77.5 °	3wt%	77.0 °
		20mol%	79.25 °	4wt%	77.6 °

Table S3. Comparison of the tensile strength

In our study		In the study by Zhao et al [17]	
reagent content	Tensile strength/MPa	reagent content	Tensile strength/MPa
0	30	0	1.36
1	29	5	3.51
2	12	10	4.14
3	9	15	5.61
		20	6.26

References

1. Zhou, H.F.; Wang, H.; Tian, X.Y.; Zheng, K.; Cheng, Q.T. Effect of 3-Aminopropyltriethoxysilane on Polycarbonate Based Waterborne Polyurethane Transparent Coatings. Prog. Org. Coat. **2014**, 77, 1073-1078.doi:10.1016/j.porgcoat.2014.03.006
2. Pergal, M.V.; Džunuzović, J.V.; Poręba, R.; Ostojić, S.; Radulović, A.; Špírková, M., Microstructure and properties of poly(urethane-siloxane)s based on hyperbranched polyester of the fourth pseudo generation. *Prog. Org. Coat.* **2013,** *76*, 743-756.10.1016/j.porgcoat.2013.01.007
3. Li, L.M.; Huang, X.A., Application of atomic force microscopy study of micro phase separation of thermoplastic polyurethane. *J. D.H.U.* **2004,** *30*, 9-13
